# Effects of Diosmin on Vascular Leakage and Inflammation in a Mouse Model of Venous Obstruction

**DOI:** 10.3389/fnut.2022.831485

**Published:** 2022-02-22

**Authors:** Junjie Zou, Dongping Yuan, Jin Yang, Yun Yu

**Affiliations:** ^1^First Affiliated Hospital of Nanjing Medical University, Nanjing, China; ^2^School of Pharmacy, Nanjing University of Chinese Medicine, Nanjing, China; ^3^Department of Vascular Surgery, Sir Run Run Shaw Hospital, Zhejiang University School of Medicine, Hangzhou, China

**Keywords:** iliac vein compression syndrome, iliac vein stenosis, venous permeability, inflammation, diosmin

## Abstract

At present, iliac vein compression syndrome (IVCS) plagues countless people, posing a significant economic and social burden. The progress of current IVCS-related research is slow owing to the limitations of animal models. In this study, we generated a mouse model of iliac vein stenosis (IVS) to monitor the effects of IVCS on venous function, such as increased vascular leakage, the expression of adhesion molecules, and elevated inflammation factors. Diosmin, a widely used clinical bioactive ingredient, was administered to confirm its therapeutic effects on the IVS mouse model. The results revealed that diosmin manifested therapeutic improvement in the IVS mouse model. In addition, we verified that the IVS mouse model is a stable and reproducible animal model for pathophysiological studies. High-purity diosmin can be beneficial to venous dysfunction and hence provides a more effective treatment option for venous diseases.

## Introduction

Iliac vein stenosis (IVS), also known as iliac vein compression syndrome (IVCS), Cockett syndrome, or May–Thurner syndrome (MTS) (1), is a disease caused by iliac vein compression (IVC). It is characterized by chronic venous insufficiency (CVI) of the lower extremities and pelvic venous reflux disturbance. The iliac vein is repeatedly stimulated by the pulsation of the iliac artery for a long time, resulting in abnormal adhesion structures in the lumen, leading to intraluminal adhesion, stenosis of the iliac vein, obstruction of venous blood flow, blood stasis of the lower extremities, and thrombosis of the iliofemoral segment (2, 3). Therefore, severe IVCS is closely related to deep venous thrombosis (DVT) and pulmonary embolism (4). The prevalence of IVCS is underestimated because most patients are asymptomatic. It has been noted that the incidence of DVT in the lower left extremity is 60% higher than that in the lower right extremity (5). In a case–control study of 230 consecutive patients with DVT by Narayan et al., they revealed that mild or moderate IVC is not associated with left DVT. However, more than 70% of the left common IVC is associated with left DVT (6). Long-term iliac vein narrowing results in venous reflux disturbance, venous hypertension of the affected lower extremity, and a series of pathological changes related to CVI of the lower extremities.

The severity of IVCS is closely related to the degree of venous hypertension, which is the hallmark of chronic venous disease (CVD) (7). Venous hypertension induces a series of pathophysiological changes in venous walls and valves (8), such as endothelial dysfunction and inflammation. This can cause microcirculatory and tissue damage and, eventually, varicose veins and venous ulcers (9). Altered venous blood flow leads to abnormal shear stress in the veins, triggering initial inflammatory responses, such as endothelial activation and leukocyte adhesion, activation, and infiltration. Over time, endothelial permeability increases, allowing red blood cells to extravasate into surrounding tissues. The obstruction of red blood cells subsequently activates mast cells and macrophages, causing them to release additional inflammatory mediators that further aggravate the deterioration, dysfunction, and inflammation of the veins (8). The type of endothelial lining determines the permeability of the vessel. In continuous microvessels, such as the lower extremities vein, the endothelial cell lining is uninterrupted (10). Although numerous studies have reported on the changes in vascular permeability in various tissues, thorough research on venous leakage of the lower extremities and its effects on surrounding tissues is still required.

In the last decade, gene-editing technology has been widely accepted and applied. Knockout mice with specific gene targets have been created to investigate disease occurrence. However, no gene-edited animals with CVD-related genes are currently available. Furthermore, owing to the lack of a mouse model of IVS, understanding IVCS has made no significant progress. Although the development of CVD is related to adhesion molecules and inflammatory factors in veins, the signaling pathways involved in this process remain unclear. Therefore, it is necessary to create a venous disease-related mouse model that can be associated with symptoms related to the onset of CVD.

Clinically, patients with CVD are often recommended to modify their lifestyles to reduce the impact of risk factors, such as sedentary behavior and prolonged standing. In addition, these patients receive veno-active drugs (VADs) or surgical treatment. Diosmin is one of the widely used therapeutic agents for VAD treatment, providing clinical benefits for patients with CVD. However, because diosmin is derived from natural plants, high impurity affects the metabolism of its active substance (11). In this study, we used diosmin of different purities in the IVS mouse model to confirm the therapeutic effects of venous diseases and examine potential molecular mechanisms.

## Materials and Methods

### Mouse Model of IVS

All animal care and experiments were performed after ethical approval from the Animal Care Committee of Nanjing University of Chinese Medicine (approval numbers ACU210305). Male C57BL/6 mice aged 8 weeks were divided into six groups, namely, sham/normal saline (NS; *n* = 3), sham/diosmin A (*n* = 3), sham/diosmin B (*n* = 3), IVS/NS (*n* = 6), IVS/diosmin A (*n* = 7), and IVS/diosmin B (*n* = 7). The doses of diosmin A (Chia Tai Tianqing Pharmaceutical, Nanjing, China) and B (Servier Tianjin Pharmaceutical Co., Ltd. Tianjin, China) were 40 mg/kg. Mice in all groups were administered the treatments intragastrically once a day.

An experimental model of IVS was created based on the model described previously (12). Under narcosis with isoflurane in a supine position, mice underwent stenosis of the left external iliac vein. A groin incision was made, and muscle tissues were separated to expose the vein. The left external iliac vein was separated from the corresponding artery while avoiding damage to the vein and artery. The left external iliac vein was ligated over a 30-G needle with a 7–0 non-absorbable suture. Subsequently, the needle was removed, and the skin was closed by 5–0 non-absorbable sutures.

### High-Performance Liquid Chromatography (HPLC)

The mobile phase of the experiment consisted of purified water, methanol, glacial acetic acid, and acetonitrile, which were mixed and sonicated. Four tablets of each of the two diosmin preparations were weighed to determine the average tablet weight. The powders were ground and weighed; dimethyl sulfoxide was added to dissolve and dilute to the mark. The mixtures were shaken well and filtered. The reference substance solution was directly used as a stock solution prepared by quality control, and the purity of the reference substance was 97.3%. The conditions of the experiment for determining the purity of diosmin preparations were as follows: column, Thermo BDS Hypersil C18 (100 × 4.6 mm, 3 μm); number, YFFX-LC-716; mobile phase, water–methanol–glacial acetic acid–acetonitrile (66:28:6:2); detection wavelength, 275 nm; injection volume, 10 μl; flow rate: 1.0 ml/min; column temperature, 35°C and running time, 10 min.

### Laser Speckle Images

Blood perfusion of the stenosis or normal hindlimb was monitored with a laser speckle imaging system (moor FLPI-2; Moor Instruments) after surgery (post), 14 and 28 days after surgery (**Figure 2A**). Mice were anesthetized continuously in a supine position with 1–1.5% isoflurane-mix gas. The region of interest was selected covering both the hindlimb paws. Perfusion was expressed as a ratio of the left (stenosis) to the right (normal) limb (13).

### Evans Blue

Mice were treated intravenously (lateral tail vein) with 0.5% Evans blue dye (#E2129, Sigma-Aldrich, Shanghai, China). If the injection was successful with the nose and skin turning blue within 10 s, Evans blue was allowed to circulate for 1 h (14). The mice were sacrificed, and intracardial perfusion was performed with NS. Subsequently, the semi-membranosus (SM) muscle tissue was dissected and weighed separately, and 500-μl formamide (#F810079, Macklin, Shanghai, China) was added to each tissue sample to incubate for 24–48 h in a 55°C water bath or heat block to extract Evans blue from tissues (15). The supernatant from the formamide/Evans blue mixture was centrifuged and measured at 620 nm. Using a standard curve of Evans blue in formamide, absorbance values were converted to the concentration (ng) of the dye.

### RNA Isolation and Real-Time PCR (RT-PCR) Analysis

The total RNA of the smooth muscle (SM) muscle was extracted using the Trizol reagent (R401-01, Vazyme, Nanjing, China), as previously described (16), and reverse-transcribed using an RT SuperMix (R323-01, Vazyme) according to the protocol of the manufacturer. Quantitative real-time PCR (qPCR) was performed on QuantStudio 5 (Applied Biosystems, Thermo Fisher Scientific) with SYBR Green Mix (Q131-02, Vazyme, China). Primers used for the related genes (interleukin-1α [IL-1α], IL-6, and monocyte chemoattractant protein-1 [MCP-1]) are listed in Supplementary Table 1. The comparative cycle time (Ct) method was used to determine fold differences between samples, and the number of target genes was normalized to the average of the sham/NS group using the 2^−Δ*ΔCt*^ method.

### ELISA Assay

The ELISA kits of mouse intracellular adhesion molecule-1 (ICAM-1, ab252355, Abcam), vascular cell adhesion molecule-1 (VCAM-1, ab201278, Abcam), alanine aminotransferase (ALT, ab282882, Abcam), aspartate aminotransferase (AST, ab263882, Abcam), and creatinine (CREA, KT21312, Mskbio) were used to detect the expression levels of ICAM-1, VCAM-1, ALT, AST, and CREA, respectively, in the serum according to the instructions of the manufacturer. Each serum sample was tested twice to limit the chance of error, and the average value was recorded.

### Statistical Analysis

All data were presented as mean ± SD. Statistical analyses were performed using GraphPad Prism 8. Differences between the two groups were compared using the unpaired Student's *t*-test for normally distributed data. Differences among groups were analyzed via one-way ANOVA analysis, followed by Tukey's multiple comparison test. Statistical significance was indicated by *p* < 0.05.

## Results

### Characteristics of Commercially Available Diosmin

**Figure 1:**
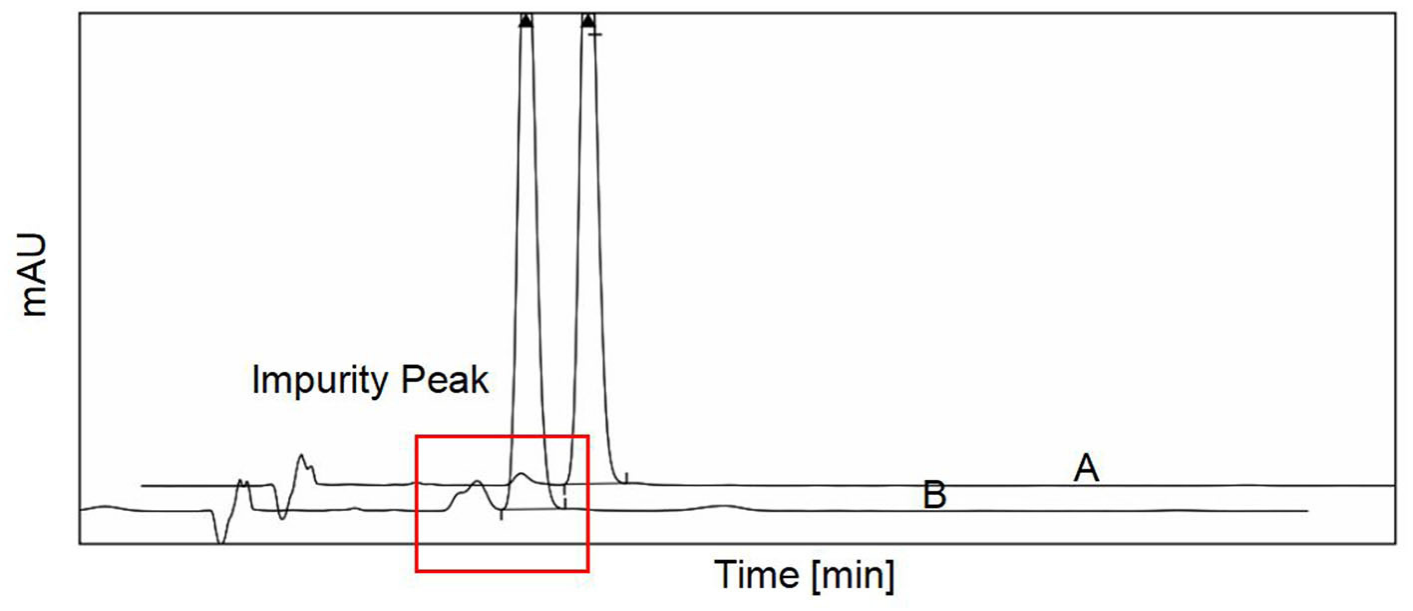
The purity of two different diosmin preparations was detected by high-performance liquid chromatography (HPLC). The two different purity of diosmin we used in this study was presented. The impurity peak was demarcated by the red frame. A, diosmin A. B, diosmin B.

The use of diosmin is recommended for the treatment of CVD according to guidelines (17). Higher-purity diosmin may have a better therapeutic effect because impurities affect the catabolism of diosmin. To verify the protective effects of diosmin on the mouse model, *in vivo* experiments were performed using two commercially available diosmin preparations. HPLC analysis was used to determine the purity of the two types of preparations, and it was observed that the impurity peak of formulation A was lower than that of formulation B (Figure 1). Following statistical analysis of the chromatographic results, it was observed that the purity of preparation A was 99%, which was higher than that of preparation B (95% purity), indicating that preparation A is more in conformity with the European Pharmacopeia^1^.

### Blood Perfusion Was Altered in the IVS Mouse Model

**Figure 2:**
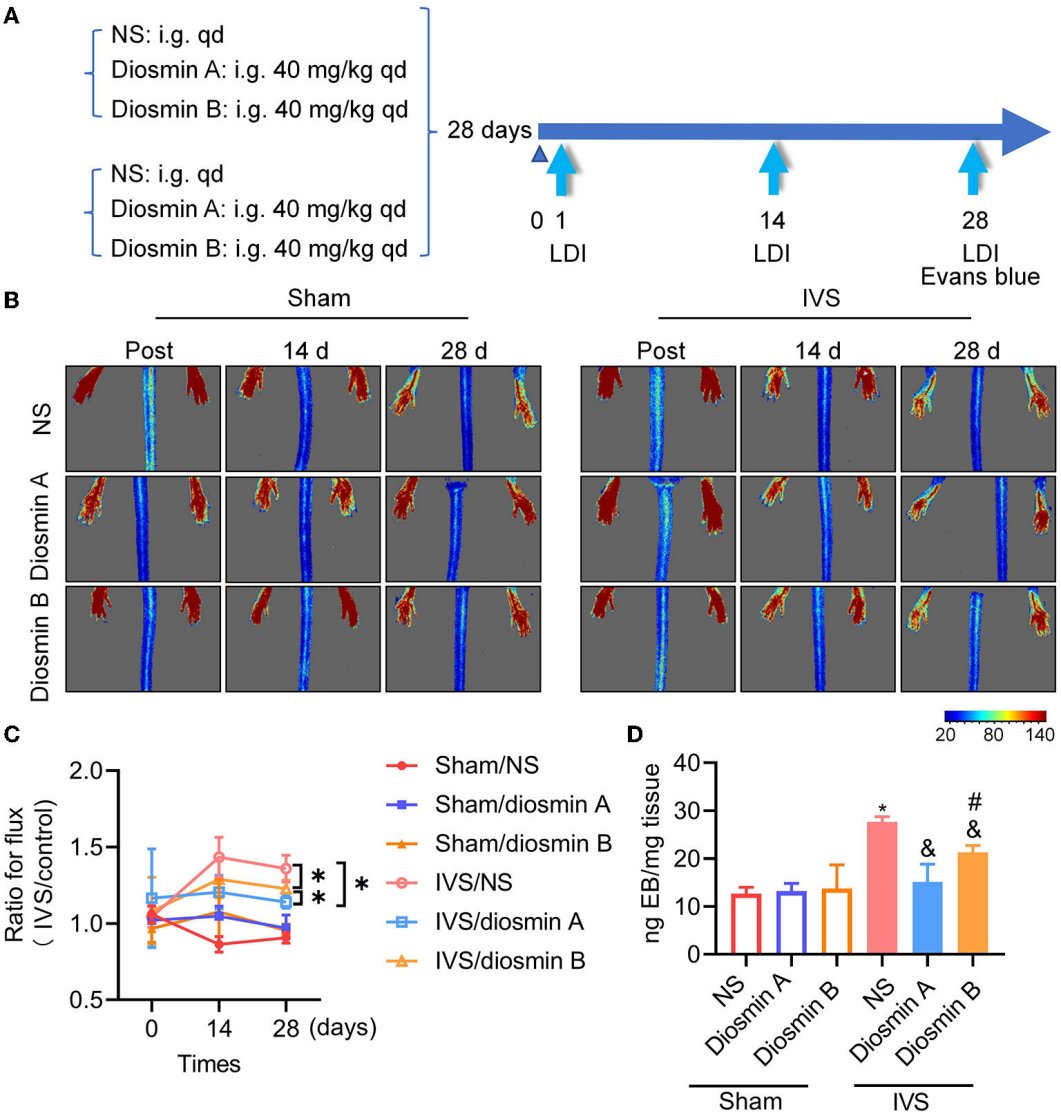
Diosmin alleviates permeability in mouse model of iliac vein stenosis. **(A)** Schema of experimental procedure. **(B,C)** The mice were detected by laser speckle on the day after surgery (post), 14 and 28 days after surgery. Sham/normal saline (NS; *n* = 3), sham/diosmin A (*n* = 3), sham/diosmin B (*n* = 3), IVS/NS (*n* = 6), IVS/diosmin A (*n* = 7), and IVS/diosmin B (*n* = 7). **(B)** The serial color-coded perfusion images were presented. **(C)** Quantification analysis of the blood perfusion achieved from laser speckle images. *p < 0.05 (Unpaired *t*-test). **(D)** Quantitative analysis of Evans blue content in semi-membranosus (SM) muscle of mice. Sham/NS (*n* = 3), sham/diosmin A (*n* = 3), sham/diosmin B (*n* = 3), IVS/NS (*n* = 6), IVS/diosmin A (*n* = 6), and IVS/diosmin B (*n* = 6). *p < 0.05 vs. sham/NS; &*p* < 0.05 vs. IVS/NS; #*p* < 0.05 vs. IVS/diosmin A (ordinary one-way ANOVA's multiple-comparisons test). IVS, iliac vein compression.

To investigate the therapeutic effects of diosmin on IVS, we employed a laser speckle imaging system to monitor the blood perfusion of the feet and toes of mice at day 1 and weeks 2 and 4 postoperatively (Figures 2A–C). On the first postoperative day, there was no significant difference in perfusion between the stenosis leg and the sham-operated leg, indicating that there was no obvious phenotype of impaired venous permeability in the acute phase (Figures 2B,C). The bilateral perfusion ratio was increased 14 days after surgery and remained high until the 28th day (*p* < 0.05). The administration of 40-mg/kg diosmin with varying purity eliminated the increase in perfusion ratio caused by stenosis and protected the vascular endothelium from damage (*p* < 0.05). Compared with diosmin B, the administration of diosmin A can better improve the bilateral perfusion ratio caused by modeling (*p* < 0.05; Figure 2C).

### Vascular Leakage Occurred in the IVS Mouse Model

Evans blue, a dye with a high affinity for albumin, was used to detect vascular permeability *in vivo*. In the IVS model, the venous endothelium was damaged owing to venous hypertension, the tight junctions of endothelial cells were destroyed and Evans blue leaked into the surrounding tissues. This was evaluated by measuring the absorbance quantification of the mouse in SM muscle, which reflected the vascular permeability *in vivo* (Figure 2D). Consistent with the results of the laser speckle image, the dye content in the IVS group was approximately twice that observed in the sham group. This finding indicated that endothelial damage led to increased permeability in the IVS model. Diosmin at a dose of 40 mg/kg protected the vascular endothelium from damage, and high-purity diosmin demonstrated a better protective effect on vascular permeability. These results indicate that the purity of diosmin can improve disease-related symptoms by inhibiting venous leakage (Figures 2C,D).

### Increased Levels of Adhesion Molecules and Inflammatory Factors in the IVS Mouse Model

**Figure 3:**
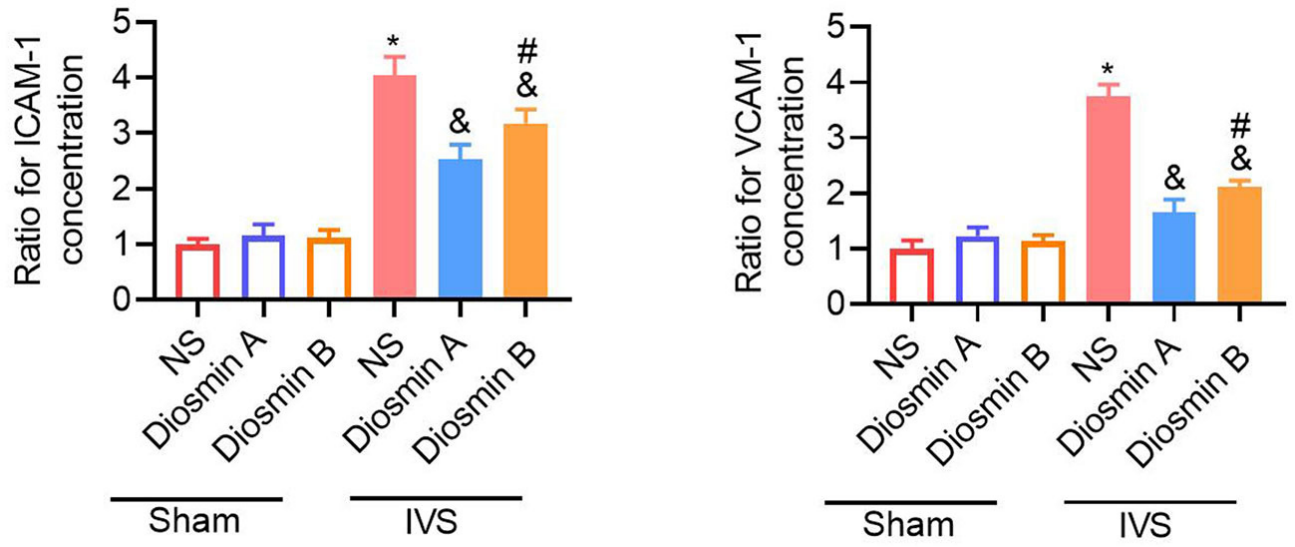
Diosmin alleviates inflammation response. The relative expression of ICAM-1 and VCAM-1 in serum was presented and normalized to the average of the sham/NS group. Sham/NS (*n* = 3), sham/diosmin A (*n* = 3), sham/diosmin B (*n* = 3), IVS/NS (*n* = 6), IVS/diosmin A (*n* = 7), and IVS/diosmin B (*n* = 7). *p <0.05 vs. sham/NS; &*p* < 0.05 vs. IVS/NS; #*p* < 0.05 vs. IVS/diosmin A (ordinary one-way ANOVA's multiple-comparisons test). IVS, iliac vein compression; ICAM-1, intracellular adhesion molecule-1; VCAM, vascular cell adhesion molecule-1.

**Figure 4:**
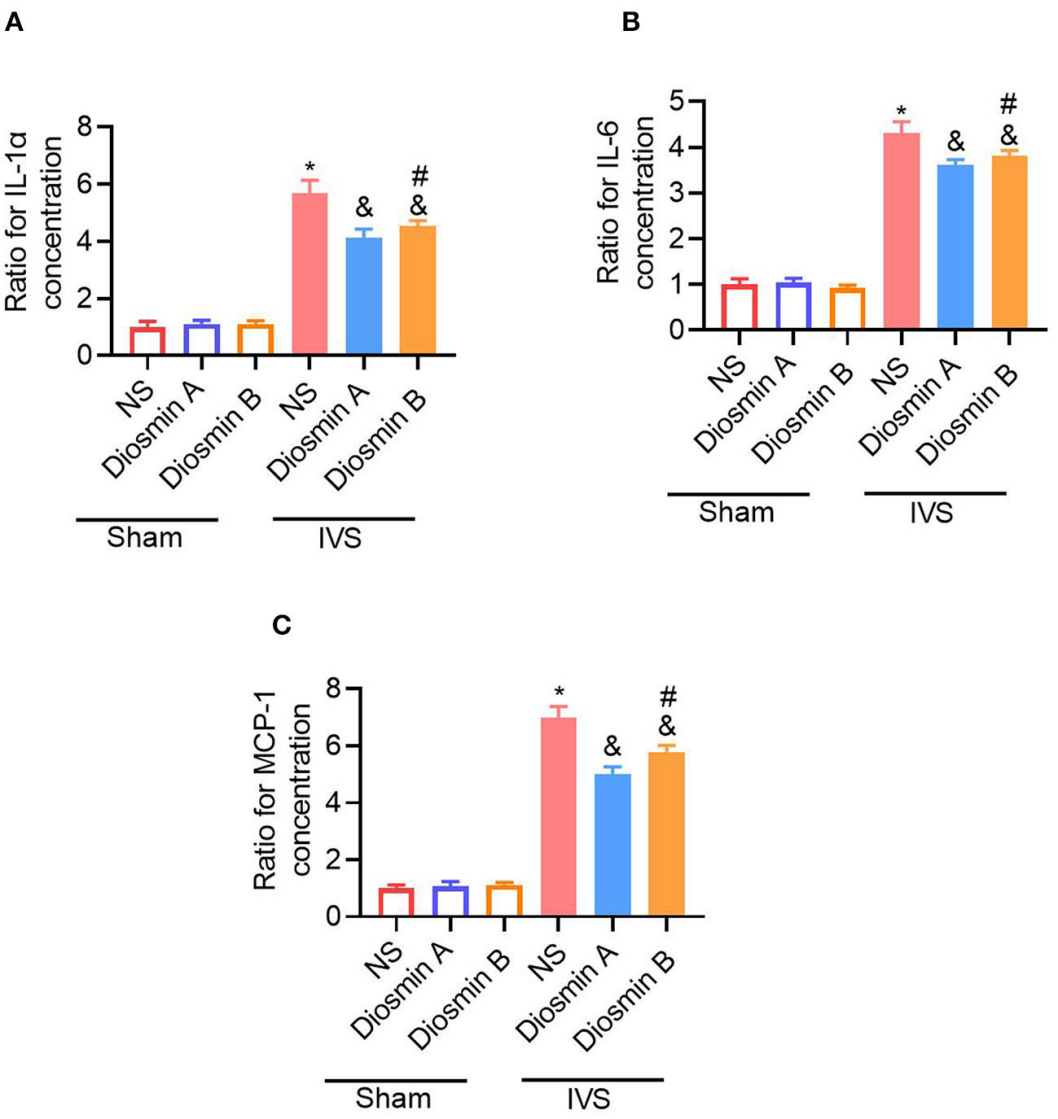
Diosmin suppresses inflammatory response in a mouse model of iliac vein stenosis. The SM muscle inflammatory cytokines IL-1α **(A)**, IL-6 **(B)**, and MCP-1 **(C)** mRNA levels were measured with quantitative real-time PCR (qRT-PCR). The number of target genes was normalized to the average of the sham/NS group using the 2^−Δ*ΔCt*^ method. Sham/NS (*n* = 3), sham/diosmin A (*n* = 3), sham/diosmin B (*n* = 3), IVS/NS (*n* = 6), IVS/diosmin A (*n* = 7), and IVS/diosmin B (*n* = 7). *p < 0.05 vs. sham/NS; &*p* < 0.05 vs. IVS/NS; #*p* < 0.05 vs. IVS/diosmin A (Unpaired *t*-test). IVS, iliac vein compression; IL-1α, interleukin-1α; MCP-1, monocyte chemoattractant protein-1.

According to previous studies, adhesion molecules play an important role in the pathogenesis of CVDs (18). Diosmin can downregulate the expression of adhesion molecules and reduce the secretion of downstream pro-inflammatory factors. In this study, the ELISA method was used to detect the expression of ICAM-1 and VCAM-1 in serum. Under physiological conditions, diosmin does not affect the levels of ICAM-1 and VCAM-1. However, under pathological conditions, diosmin significantly decreased the ICAM-1 and VCAM-1 levels caused by stenosis, and high-purity diosmin improved the levels of adhesion molecules (*p* < 0.05; Figure 3). In addition, we further investigated the expression of inflammatory factors, such as IL-1, IL-6, and MCP1. qRT-PCR revealed that the mRNA expression of the inflammatory factors IL-1, IL-6, and MCP-1 was increased after stenosis (*p* < 0.05; Figures 4A–C). Both drugs at the dose of 40 mg/kg significantly decreased the mRNA expression of inflammatory factors (*p* < 0.05). Diosmin A significantly improved the expression of inflammatory factors when compared to diosmin B (*p* < 0.05).

### Diosmin Did Not Cause Kidney or Liver Damage

**Figure 5:**
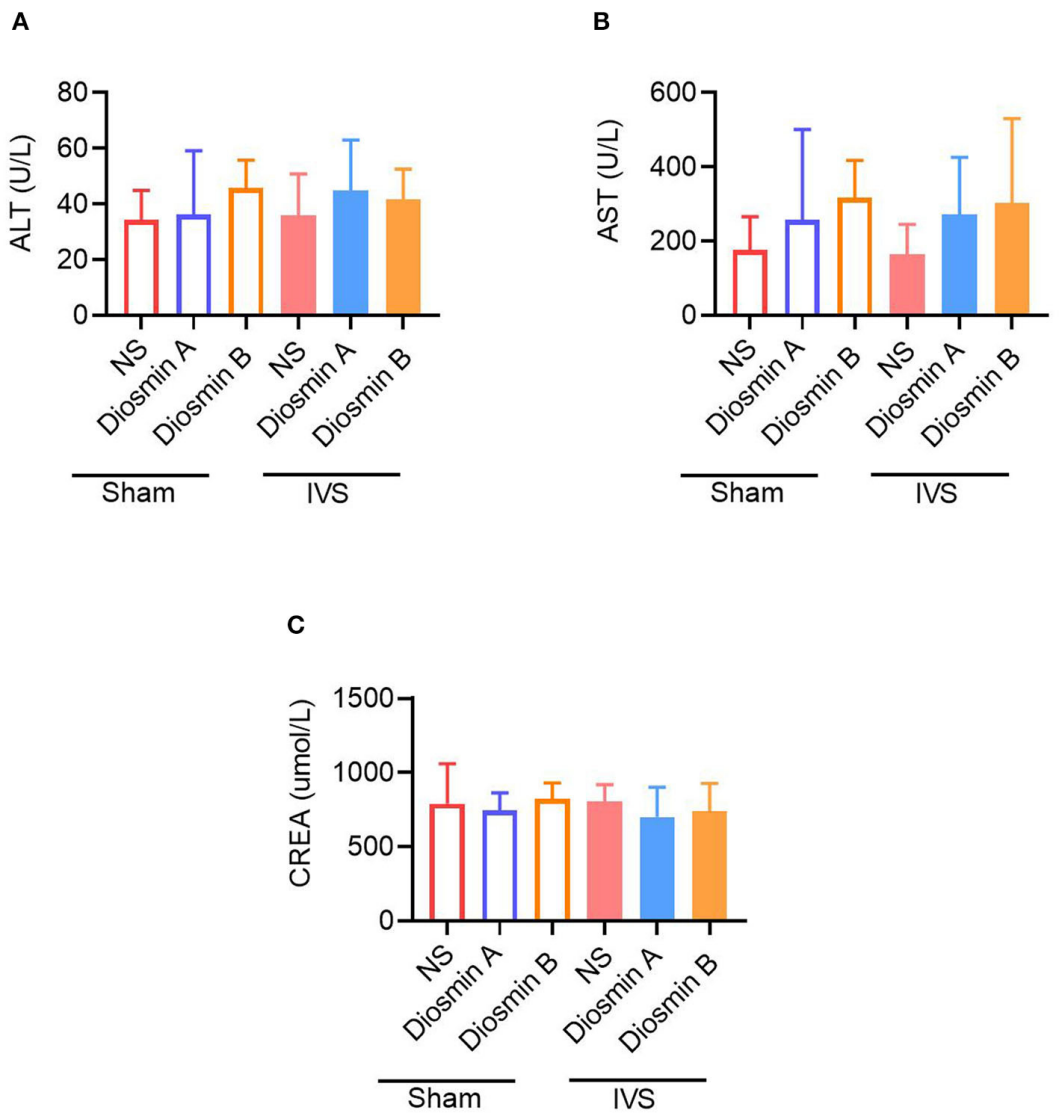
The high purity of diosmin has no liver and kidney damage. The content of liver function index alanine aminotransferase (ALT) **(A)** and aspartate aminotransferase (AST) **(B)** renal function index creatinine (CREA) **(C)** in mice after administration of different purity of diosmin. Sham/NS (*n* = 3), sham/diosmin A (*n* = 3), sham/diosmin B (*n* = 3), IVS/NS (*n* = 6), IVS/diosmin A (*n* = 7), and IVS/diosmin B (*n* = 7). IVS, iliac vein compression.

Although the abovementioned results suggest that high-purity diosmin plays a key role in maintaining the venous function, whether it causes kidney or liver injury remains unclear. Therefore, we tested the liver and kidney function in the serum of mice at 28 days after the operation (Figures 5A–C). The results revealed that after the administration of diosmin of varying purity, no significant difference was observed in the liver function indices ALT and AST and the renal function index CREA in mice. There was no significant change when compared to the non-administration groups. This finding confirmed that high-purity diosmin does not cause kidney or liver injury, which supports the selection of suitable pure drugs in clinical settings.

## Discussion

Chronic venous disease, which causes fatigue, pain, and oedema in the lower limbs, is mainly caused by abnormal venous structure and valves, insufficient venous blood return to the heart, and excessive venous hypertension (19). The main clinical manifestations for IVCS include varicose veins, skin nutritional changes, and venous ulcers. In China, the prevalence of venous diseases of the lower extremities has increased to 8.89%, and approximately 100 million patients have been affected. Every year, a new incidence rate of venous diseases emerges, ranging from 0.5 to 3.0%, with venous ulcers accounting for 1.5% (20). At the early stage of CVD, the dysfunction of the venous valve causes a backflow of blood, increasing persistent venous pressure. Venous hypertension leads to the hydrostatic pressure of superficial subcutaneous veins and capillaries, resulting in capillary filtration and tissue oedema, thereby, reducing the quality of life of patients and increasing the burden of society (21). Numerous factors are responsible for superficial varicose veins in the lower extremities, such as primary venous valve insufficiency and secondary DVT of the lower extremities, the defects of congenital venous valve function, and other IVCSs.

During the process of IVS, both venous hypertension and blood accumulation can further expand the diameters of the vein and injure the venous valves. At the beginning of IVS, venous endothelial cells are injured by venous hypertension, which activates white blood cells in circulation and causes a reduction in L-selectin and CD11b in white blood cells (22, 23). In addition, the levels of inflammatory factors, such as the soluble L-selectin, adhesion molecules ICAM-1, endothelial leukocyte adhesion molecule-1, and VCAM-1, are increased in plasma (24, 25). These inflammatory responses increase endothelial cell adhesion and infiltration into local tissues and further aggregate the inflammatory response in platelets and monocytes, resulting in the production of more inflammatory mediators and cell adhesion-related factors, thereby prolonging chronic inflammation response. With the development of IVS, a “fibrin cuff” is formed around the tortuous and expanded capillaries, impeding the diffusion of oxygen. In addition, chronic inflammation causes the accumulation of matrix metalloproteinase, resulting in endothelial dysfunction, accompanied by excessive degradation of the extracellular matrix (26). Substantially, this response promotes the formation of skin dystrophic lesions (pigmentation) and ulcers in the boot area. Therefore, chronic inflammation and endothelial dysfunction in veins play a vital role in the occurrence and progression of IVS (27).

Currently available animal models related to CVDs mainly include the rat unilateral femoral vein ligation venous hypertension model and the mouse unilateral femoral artery ligation hindlimb ischemia model. Owing to their large size, it is easy to perform surgery on rats. However, most *in vivo* models are established in mice owing to the availability of transgenic mice. Therefore, we decided to conduct this study using a reproducible mouse model. Although the study results of the mouse artery model can represent the response of blood vessels to a certain extent, the structural variations between arteries and veins make it difficult to generalize the results to veins. The mouse model of IVS that we constructed can be used to develop novel disease models. Because mice and humans share similar genes, relevant research can help to predict the emergence and development of human diseases to a certain extent. This model can simulate the related CVD symptoms caused by human IVS, which is helpful for the exploration of various types of CVD-related diseases and the development of new drug intervention targets in the future. Finally, by constructing gene-edited animals, the occurrence and development of diseases can be interpreted from the perspective of genes, allowing for more research studies on disease mechanisms.

Nowadays, the non-surgical treatment of CVD remains an effective therapeutic strategy that can be used for varicose vein treatment. In China, surgery remains a major therapeutic strategy for high ligation of the great saphenous vein/stripping. Recently, the concept of minimally invasive treatment has been widely accepted, and its efficacy is comparable to that of high saphenous vein ligation/stripping. It is not possible to cure CVD with only surgical treatment. Therefore, non-surgical treatment plays an essential role in alleviating the symptoms of the disease after surgery. Diosmin is a flavonoid compound extracted from natural plants. It has been reported that diosmin plays multiple roles in venous protection, such as anti-inflammatory response, alleviating endothelial cell activation, and leukocyte adhesion. Hesperidin is often accompanied by diosmin as an impurity (11). According to the United States Pharmacopeia, the impurities of other flavonoids, such as hesperidin in diosmin preparations, should be strictly controlled within 10%. However, whether these impurities affect the efficacy of diosmin remains unclear. Previous studies have demonstrated that these flavonoids have pharmacological activity (28). In clinical settings, the content of these compounds is far from the therapeutic dose reported in animal studies. In this study, we observed that the purity of diosmin plays a key role in the vein and muscle injury caused by IVS. Mechanically, diosmin eliminated the increase of foot perfusion rate caused by stenosis to some extent, protected vascular endothelial cells from injury, reduced venous leakage, and maintained the tight junction of muscle cells around blood vessels. In addition, diosmin reduced the inflammatory response induced by IVS. These results are consistent with the use of diosmin in the treatment of venous dysfunction-related diseases in clinical practice. Importantly, high-purity diosmin has a better therapeutic impact.

Drug safety has always been regarded as a critical concern of both clinicians and patients. It has been reported that more than 3,000 mg/kg of diosmin is lethal (29), which is 75 times the daily therapeutic dose used in this study. In a study on the safety and security of Daflon, Meyer et al. revealed that the drug was completely eliminated after 96 h of administration in rats with no untoward accumulation in any specific organs (29). This finding can explain why diosmin did not cause liver or kidney damage in this study.

Our results revealed the effects of diosmin of different purity on vascular leakage in mice with IVS and provided a theoretical basis for the selection of diosmin clinically. However, owing to the limitations of the mouse model, there may be other reasons for the variation in efficacy of the two types of diosmin preparations. Because the number of mice used in this experiment was insufficient, further studies should be conducted. In addition, a single dose of diosmin A and B was selected; however, it is more convenient to evaluate the therapeutic effect of drugs using a series of drug concentration gradients. Because diosmin is a widely used VAD, research on its purity should be integrated with statistical analysis of clinical big data to yield more compelling results.

In conclusion, the purity of diosmin plays a key role in vein and muscle injury caused by IVS. Diosmin eliminated the increase in foot perfusion rate caused by stenosis to some extent, protected vascular endothelial cells from injury, reduced venous leakage, and maintained the tight junction of muscle cells around blood vessels in mice. In addition, diosmin alleviated the inflammatory response induced by IVS, and high-purity diosmin had a better therapeutic impact. Therefore, in-depth studies on the mechanism of diosmin in IVS and the effects of diosmin of different purity may provide a reference for clinical drug use.

## Data Availability Statement

The original contributions presented in the study are included in the article/Supplementary Material, further inquiries can be directed to the corresponding author/s.

## Ethics Statement

The animal study was reviewed and approved by Animal Care Committee of Nanjing University of Chinese Medicine.

## Author Contributions

YY and JY designed the experiments. DY and JZ performed the experiments, analyzed the data, and wrote the manuscript. YY and JZ analyzed the data and revised the manuscript. All authors contributed to the article and approved the submitted version.

## Funding

This study was supported by grants from the National Natural Science Foundation of China (grant number 81973823).

## Conflict of Interest

The authors declare that the research was conducted in the absence of any commercial or financial relationships that could be construed as a potential conflict of interest.

## Publisher's Note

All claims expressed in this article are solely those of the authors and do not necessarily represent those of their affiliated organizations, or those of the publisher, the editors and the reviewers. Any product that may be evaluated in this article, or claim that may be made by its manufacturer, is not guaranteed or endorsed by the publisher.

## Footnotes

^1^*European Pharmacopoeia*. Available online at: https://pheur.edqm.eu/home.
