# Supplementary material for: Effects of Diosmin on Vascular Leakage and Inflammation in a Mouse Model of Venous Obstruction
Source: Front Nutr. 2022 Feb 22;9:831485. doi: 10.3389/fnut.2022.831485 (PMC8903897; doi:10.3389/fnut.2022.831485)
Supplement: Supplementary file 1 [file Table_1.docx]

Table 1

|  | Gene | Forward | Reverse |
| --- | --- | --- | --- |
| mouse | IL -1a | CGAAGACTACAGTTCTGCCATT | GACGTTTCAGAGGTTCTCAGAG |
| mouse | IL-6 | TCTATACCACTTCACAAGTCGGA | GAATTGCCATTGCACAACTCTTT |
| mouse | MCP-1 | GCATCCACGTGT TGGCTC | CTCCAGCCTACTCATTGGGATCA |

Supplementary Table 1. The primers used for mRNA quantitative real-time PCR (qRT-PCR) assays. Interleukin-1a, IL-1a; interleukin-6, IL-6; monocyte chemoattractant protein-1, MCP-1.
